# Patient-Derived Organoid Biobanks for Translational Research and Precision Medicine: Challenges and Future Perspectives

**DOI:** 10.3390/jpm15080394

**Published:** 2025-08-21

**Authors:** Floriana Jessica Di Paola, Giulia Calafato, Pier Paolo Piccaluga, Giovanni Tallini, Kerry Jane Rhoden

**Affiliations:** 1 Biobank of Research, IRCCS Azienda Ospedaliero-Universitaria di Bologna, 40138 Bologna, Italy; florianajessica.dipaola@aosp.bo.it (F.J.D.P.); pierpaolo.piccaluga@unibo.it (P.P.P.); 2 Department of Medical and Surgical Sciences (DIMEC), University of Bologna, 40138 Bologna, Italy; giovanni.tallini@ausl.bologna.it (G.T.); kerry.rhoden@unibo.it (K.J.R.); 3 Solid Tumor Molecular Pathology Laboratory, IRCCS Azienda Ospedaliero-Universitaria di Bologna, 40138 Bologna, Italy

**Keywords:** organoids, living biobanks, translational research, personalized medicine

## Abstract

Over the past decade, patient-derived organoids (PDOs) have emerged as powerful in vitro models that closely recapitulate the histological, genetic, and functional features of their parental primary tissues, representing a ground-breaking tool for cancer research and precision medicine. This advancement has led to the development of living PDO biobanks, collections of organoids derived from a wide range of tumor types and patient populations, which serve as essential platforms for drug screening, biomarker discovery, and functional genomics. The classification and global distribution of these biobanks reflect a growing international effort to standardize protocols and broaden accessibility, supporting both basic and translational research. While their relevance to personalized medicine is increasingly recognized, the establishment and maintenance of PDO biobanks remain technically demanding, particularly in terms of optimizing long-term culture conditions, preserving sample viability, and mimicking the tumor microenvironment. In this context, this review provides an overview of the classification and worldwide distribution of tumor and paired healthy tissue-specific PDO biobanks, explores their translational applications, highlights recent advances in culture systems and media formulations, and discusses current challenges and future perspectives for their integration into clinical practice.

## 1. Introduction

The development of intestinal organoid culture technology in 2009 marked a major milestone in organ development research and preclinical cancer models [1]. In recent decades, preclinical cancer biology research has frequently utilized immortalized two-dimensional (2D) cancer cell lines in laboratory settings, along with xenografted or transgenic animal models. While these methods have provided significant insights, continuously passaged cell lines may not faithfully reflect the biology of the original parental tumors, and the development and utilization of animal models can be both expensive and time-consuming. To reduce the gap between the limitations of traditional cell culture models and the complexity of living tissue, Sato and colleagues utilized rapidly proliferating Lgr5+ adult stem cells, found at the base of mouse intestinal crypts, to pioneer a controlled three-dimensional (3D) ex vivo culture system. By combining extracellular matrix components with a variety of growth factors, they partially recreated the healthy tissue or tumor niche closely resembling in vivo conditions while also retaining patient-specific characteristics. This innovation led to the formation of “mini-intestines” featuring a consistent villus–crypt structure and a full range of specialized cell types. The system was later adapted to produce human intestinal organoids, and extended to other Lgr5+ stem cell-containing organs, including the colon, stomach, and liver [2,3,4], and other non-Lgr5+ stem cell populations, such as those of lung, pancreas, and endometrium [5,6,7].

**Figure 1:**
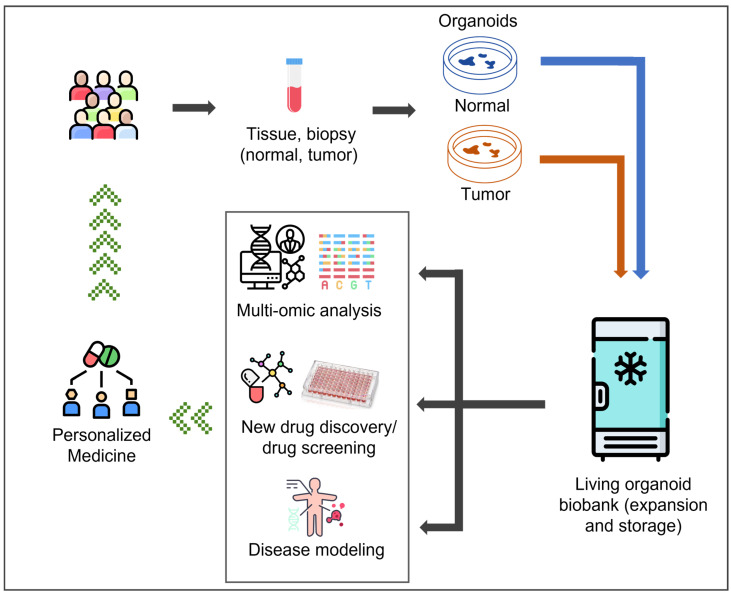
Establishment of a living organoid biobank and its applications in personalized medicine.

Over the past decades, organoids—3D cell culture models—have been generated from patients’ own tumor or healthy tissues (patient-derived organoids, or PDOs) originating from various organs and body regions. Several omics studies have confirmed that, as opposed to monolayered primary or immortalized cell lines, PDOs more closely resemble parental tissue as they: (i) recapitulate tissue-specific histological features; (ii) preserve the full spectrum of differentiated cell types and stem-cell hierarchy; (iii) maintain disease-associated genetic mutations and related drug response; and (iv) exhibit cell–cell and cell–matrix interactions, generating physiological gradients of oxygen, nutrients and growth factors and thus replicating organ-level processes like barrier development, secretion, and metabolic zonation [8,9,10,11,12]. While most of the existing literature is focused on the tumor-derived PDO used in translational research, the collection of paired (or unpaired) healthy tissue-specific PDOs, mainly used as the “control group”, has been fundamental for improving our knowledge of tumor onset and progression as well as in chemoprevention studies. These features make PDOs, either from tumor or paired healthy specimens, the most reliable in vitro model for functional analyses, personalized therapies, drug-response studies for prediction medicine, and disease modeling in translational research. Indeed, PDOs are suited to multi-omic approaches that, in conjunction with clinical data, can unveil predisposition, prognostic, predictive and diagnostic biomarkers to provide personalized care for specific diseases [13,14]. (Figure 1). Predicting who will benefit most from specific therapies represents the main challenge of many preclinical studies.

**Figure 2:**
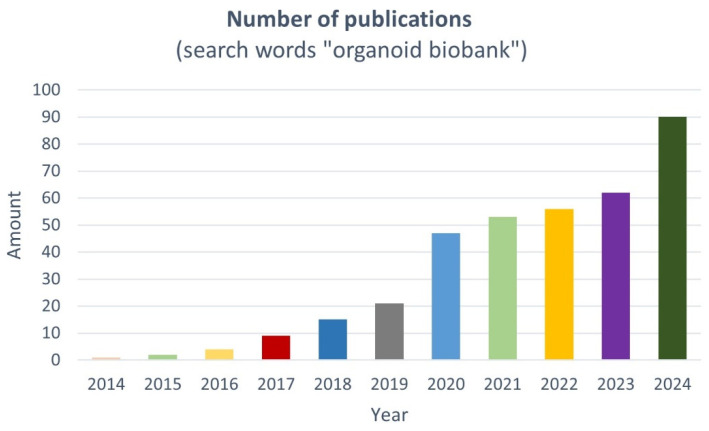
Number of papers published per year on organoid biobanks since 2014, according to PubMed. Search words: organoid biobank (databases: PubMed, Google Scholar).

During the last decade, researchers and clinicians have created living tumor and paired healthy tissue-specific PDO biobanks, which, in conjunction with patient-specific clinical information, offer a unique opportunity to establish repositories of diverse physiologically relevant disease models (Figure 1 and Figure 2 and Table 1) [15,16]. These models can be used worldwide for a range of applications, including multi-omic approaches, drug development and screening, disease modeling, and clinical implementation of precision medicine (Figure 1).

Nowadays, PDOs are the most reliable cost- and time-efficient pre-clinical model available. However, establishing and maintaining PDO biobanks remain technically challenging, particularly in optimizing cell culture conditions, enhancing experimental reproducibility, preserving sample viability, and mimicking the tumor microenvironment.

In this study, we present a comprehensive classification of living tumor and paired healthy tissue-specific PDO biobanks worldwide, examining their diverse applications in translational cancer research and personalized medicine. We highlight recent advances in culture systems and media formulations that have improved the maintenance and expansion of PDOs. Furthermore, we address current challenges—such as sample acquisition, quality control, and the standardization of culture procedures—and discuss future directions to enhance the utility of PDO biobanks in clinical settings, particularly in advancing precision medicine.

## 2. Living PDO Biobanks: Classification and Worldwide Distribution

Traditional biobanks collect non-regenerative or static biospecimens and related clinical data that can be primarily used in omics observational studies to distinguish diseased from healthy patients [15]. However, the growing need to uncover the molecular mechanisms underlying tumor development and progression and to develop new targeted drug therapies calls for the use of regenerative biospecimens. In this context, preserving living biobanks and collecting highly self-renewing tissues suitable for the establishment of tridimensional (3D) models, able to respond to the need of translational and personalized medicine, is vital [15]. This has led to the widespread creation of 3D living biobanks (or next-generation biobanks), aside from traditional ones, all over the world [16]. Among the 3D models, patient-derived organoids (PDOs), derived from either adult/fetal progenitor stem cells (ASC/FSC) of healthy and tumor and paired healthy primary donor tissue or from induced pluripotent stem cells (iPSCs), represent one of the most valuable ones [55]. Indeed, substantial evidence shows that PDOs retain the genetic and histological features of parental tissue (or the specific characteristic of the desired tissue when derived from single ASC/FSC or iPSCs) [56].

Since the first PDOs were established from mouse small intestine in 2009 [1], numerous other PDOs have been developed from various types of human primary tumors and paired normal tissues [2,46,50,57,58,59].

To date, five major academic and commercial living PDO biobanks have been established to cover a comprehensive range of tumor types (including paired healthy tissue): the academic biobank from Hubrecht Institute (Utrecht University Medical Center and the Royal Netherlands Academy of Arts and Sciences) and the commercial biobanks from Sigma–Aldrich, the American Typical Culture Collection (ATCC), and Cellesce and DefiniGEN [60,61,62,63,64]. These biobanks collect PDOs from a variety of tumor types originating either from donor primary tumors and matched healthy tissue, or from iPSCs.

**Figure 3:**
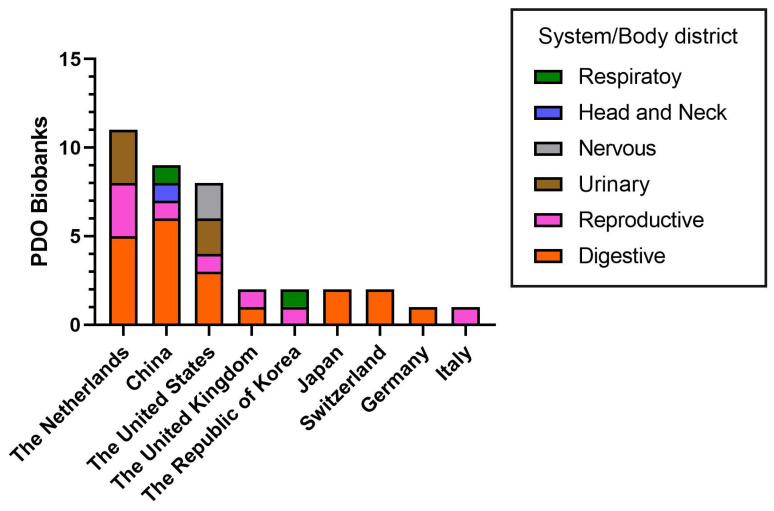
Worldwide distribution of tumor-specific patient-derived organoid biobanks. Search words: organoid, tumor patients derived organoid, biobank (databases: PubMed, Google Scholar).

In addition, several tumor-specific living PDO biobanks have been created for tumors—and paired healthy tissue—of digestive (*n* = 20), reproductive (*n* = 8), urinary (*n* = 5), nervous/head and neck (n = 3) and respiratory (*n* = 2) systems. Of these, digestive (20/38, 52.6%) and reproductive system (8/38, 21.1%) PDO biobanks are the most represented (Figure 3).

The main features characterizing PDO biobanks are safety, accuracy and convenience. The correct management of a biobank requires specific documentation of protocols and processes and specialized personnel to manage samples. In addition, sample handling, the processing protocols to establish PDOs, optimization of PDO culture media, and the preservation and storage of PDOs in biobanks require special attention.

The majority of living PDO biobanks are located in the Netherlands (11/38, 28.9%), China (9/38, 23,6%) and the United States (8/38, 21.1%), with fewer located in the United Kingdom (2/38, 5.3%), the Republic of Korea (2/38, 5.3%), Japan (2/38, 5.3%), Switzerland (2/38, 5.3%), Germany (1/38, 2.6%) and Italy (1/38, 2.6%) (Figure 3).

A comprehensive overview of tumor and paired healthy tissue-specific PDO biobanks—including their key features, the principal findings of translational research, and country of reference—is reported in Table 1.

## 3. Living PDO Biobanks: Translational Research and Personalized Medicine

Thanks to their intrinsic ability to recapitulate multiple features of the parental tissue, PDOs are powerful tools for translational research and to develop targeted-drug therapy for precision and personalized medicine. Indeed, PDOs are extremely important disease models to study the molecular mechanisms associated with tumor development and progression, therapy resistance and tumor recurrence, as well as for high throughput screening to select the most sensitive drug (especially in refractory/metastatic patients) and discover new targeted-drugs [56,65].

Here, we report some of the key findings of translational research studies involving tumor and paired healthy tissue-specific PDO biobanks, subdivided by anatomical system or body district (Table 1).

### 3.1. Digestive System

Several PDO biobanks have been created for digestive cancers, including gastrointestinal, liver, pancreatic and neuroendocrine tumors.

In 2015, Hans Clevers’ group pioneered the establishment of the first colorectal cancer (CRC) PDO biobank, demonstrating that the somatic genetic alterations and gene expression profiles of PDOs matched those of the corresponding primary CRC tissues. In addition, high-throughput drug screening showed that *RNF43*-mutated PDOs were more sensitive to Wnt inhibitors compared to *APC*-mutated ones, revealing a strong gene–drug response correlation [17].

Since then, several other digestive tumor-PDO biobanks have been developed using samples from treated or untreated patients with gastrointestinal, liver, and pancreatic neuroendocrine tumors with different staging, grading and molecular subtypes [18,29,33,36]. These biobanks have been mainly used to confirm the ability of organoids to recapitulate the genetic features of primary tissue, to unveil the molecular mechanisms underlying tumor onset and progression, for high-throughput drug screening in gene–drug response correlation studies and to enable the use of PDOs as a predictive model of patient response in the clinic.

#### 3.1.1. Gastrointestinal Tumors

Several studies have highlighted the importance of gastrointestinal tumor-PDO biobanks for disease modeling in the evaluation of molecular mechanisms associated with tumor development and progression. Fujii and colleagues established a CRC-PDO biobank from a range of histological subtypes and clinical stages, demonstrating that mutations acquired during the adenoma–carcinoma transition of organoids correlated with a niche factor-independent growth [18]. Molecular analysis of an early-onset colorectal cancer (EOCRC)-PDO biobank has shown that organoids with RSPO fusions resembled the features of normal organoids and were distinct from *APC*-mutated organoids, with high BMP2 and low PTK7 expression [19]. An interesting study performed on a biobank of primary CRC-PDOs and paired peritoneal metastasis organoids (PMPO) unveiled that primary CRC-PDOs and PMPO belonged to the Consensus Molecular Subtype 4 (CMS4) and were resistant to oxaliplatin because of the high levels of glutamate-cysteine ligase causing detoxification of oxaliplatin through glutathione synthesis [22]. Large-scale functional screening of a heterogeneous CRC-PDO biobank and paired healthy colonic mucosa samples resulted in the generation of MCLA-158, a dual-targeting bispecific antibody that induces EGFR degradation in leucine-rich repeat-containing G-protein-coupled receptor 5-positive (LGR5+) cancer stem cells with minimal toxicity toward healthy LGR5+ colon stem cells [25]. In a CRC-PDO (Stage I-IV) biobank, gene–drug response studies showed that *RAS/RAF* mutations serve as a signature for predicting sensitivity to cetuximab (an EGFR inhibitor) [26]. Moreover, large-scale drug screening performed on gastrointestinal tumor-PDOs of all molecular subtypes, including Epstein Barr virus (EBV), microsatellite instability (MSI), intestinal/chromosome instability (VIN), and diffuse/genomic stable subtypes, revealed unexpected sensitivity to several drugs, including napabucasin, abemaciclib, and the ATR inhibitor VE-822 [28].

The ability of PDOs to identify signatures that predict patient response in the clinic has been shown in several studies, using metastatic chemo-refractory gastrointestinal tumor-PDO biobanks [27], locally advanced rectal cancer-PDO biobanks from patients treated with neoadjuvant chemoradiation [20], advanced CRC-PDO biobanks [21,24] and biobanks of liver metastasis-PDOs from CRC patients [23]. Interestingly, Schumacher and colleagues investigated a cohort of well-characterized CRC PDOs using targeted proteomics and identified heterogeneous activation of the mitogen-activated protein kinase (MAPK) signaling pathway, which contributed to differential responses to EGFR inhibition [66].

#### 3.1.2. Liver, Pancreatic and Neuroendocrine Tumors

The establishment of a liver carcinoma PDO biobank allowed researchers to demonstrate that organoids derived from heterogeneous hepatocellular carcinoma (HCC) retain the histopathological and somatic genetic alterations of the originating tumor and exhibit differential drug responses to sorafenib, a multiple tyrosine kinase inhibitor [29].

Pancreatic tumor PDO biobanks have been used to identify the molecular mechanisms associated with tumorigenesis. Beato and colleagues developed a biobank of PDOs derived from intraductal papillary mucinous neoplasm (IPMN), a precursor of pancreatic cystic tumors, which recapitulated the histologic and genomic features of the parental IPMN, thereby providing a valuable model to study IPMN biology and its progression to invasive carcinoma [30]. Another study identified 28 genes differentially expressed between normal pancreatic duct and IPMN organoids, with *CLDN18* and *FOXA1* genes showing the most significant upregulation and downregulation, respectively. Immunohistochemical analysis strengthened the idea that the loss of *FOXA1* expression occurred early in pancreatic tumorigenesis [33].

A pancreatic ductal adenocarcinoma (PDAC)-PDO biobank from cachectic and non-cachectic patients allowed for the identification of tumor-derived factors driving cancer cachexia. Specifically, certain cachexia-related markers (LIF, IL-8, and GDF15) were significantly elevated in PDAC-PDOs from cachectic compared to non-cachectic patients, while others (IL-1α and IL-1β) were reduced [35].

Other studies have highlighted the potential of PDO biobanks to investigate gene–drug associations and to predict the chemotherapeutic response of patients with pancreatic tumors [31,34]. Using a PDAC-PDO biobank, missense—but not frameshift—mutations in the PDAC driver gene *ARID1A* were found to be associated with increased sensitivity to the kinase inhibitors dasatinib and VE-821. PDAC-PDOs were also sensitive to 26 additional compounds, most of which are currently approved for other types of tumors [31]. High-throughput screening of 76 therapeutic agents against a PDAC-PDO biobank revealed drug sensitivities previously not exploited in the clinic. In particular, the PRMT5 inhibitor EZP015556 was shown to target pancreatic tumors negative for *MTAP* (a gene commonly lost in pancreatic cancer), as well as a subset of *MTAP*-positive tumors [34]. Using a heterogeneous biobank of PDAC-PDOs from chemotherapy or radiation-treated and untreated patients, a correlation was identified between PDO chemotherapy sensitivity and patient therapeutic response—particularly to oxaliplatin—demonstrating the potential of PDO biobanks to predict clinical drug responses [32].

Kawasaki and co-workers created a PDO biobank from gastroenteropancreatic (GEP) neuroendocrine neoplasms, including neuroendocrine tumors (NEN) and neuroendocrine carcinomas (NEC). Both NEN-PDOs and NEC-PDOs recapitulated the pathohistological and functional phenotypes of the parental tumors. Interestingly, NEN-PDOs are characterized by chromosome-wide loss of heterozygosity, expression of specific transcription factors such as NKX2-5 and independence from Wnt and EGF niche factors (irrespective of genetic mutations) [36].

### 3.2. Reproductive System

Numerous PDO biobanks have been created for reproductive cancers, mostly for breast carcinoma and fewer for ovarian and cervical tumors. Currently, the choice of therapeutic strategy for breast cancer relies on the differential expression of estrogen receptor (ER), progesterone receptor (PR) and epidermal grown factor receptor (HER2). One of the first breast-PDO biobanks was established from HER2-positive, HER2-negative and triple negative breast cancer patients (TNBC), showing an acceptable degree of concordance between the primary tumor and PDO histology [39]. A few years later, a breast-PDO biobank from primary breast tumors (including TNBC, ER+/PR+ and Her2+) was used to optimize highly versatile protocols for long-term culture, genetic manipulation and xenotransplantation of organoids in mice [37]. An interesting study elucidated several molecular features characterizing primary and metastatic TNBC. A TNBC-PDO biobank was used to validate four molecular consensus subtypes (CMS), stem-like, mesenchymal-like, immunomodulator, and luminal-androgen receptor. Each of them was characterized by a distinct tumor-associated microenvironment and pathway activation pattern, and a CSM-dependent drug response that matched those of patients in the clinic [41]. Another study reported the establishment of a TNBC-PDO biobank for disease modeling, demonstrating that long term-cultured PDOs had specific signatures of aggressive *MYC*-driven basal-like breast cancers and were largely comprised of luminal progenitor (LP)-like cells with hyperactivation of *NOTCH* and *MYC* signaling [42]. The predictive potential of PDOs for patient drug responses was evaluated using a breast-PDO biobank from primary and metastatic breast carcinomas that recapitulated the morphogenetic heterogeneity and gene expression of parental tumors. Indeed, high-throughput drug screening revealed that the drug response of PDOs matched that of corresponding patients or xenotransplanted mice, especially for tamoxifen [38]. Similar results were obtained using a breast cancer-PDO biobank from neoadjuvant-chemotherapy treated patients, showing that PDOs could predict the clinical response of patients to neoadjuvant chemotherapy [40].

Cervical carcinoma PDO biobanks, established from normal or Pap-brush cervical tumor tissues, have been used to model sexually transmitted viral infections, highlighting their role in tumor carcinogenesis and differential drug response [43]. Another group created an ovarian cancer-PDO biobank, enabling a better understanding of CIN, genome evolution and tumor micro-heterogeneity [44].

### 3.3. Urinary System

PDO biobanks were also created for tumors of the urinary system, including kidney, bladder and prostate cancers. In 2020, the first pediatric PDO biobank was established for kidney tumors (including Wilms tumors, rhabdomyoma, renal cell carcinoma, and congenital mesodermal nephroma) that exhibited similar disease features and tissue heterogeneity [45]. Two independent groups established bladder cancer-PDO biobanks and demonstrated that PDOs retained the tumor heterogeneity of parental tissue and showed only a partial correlation between drug response and mutational profile [46,47]. Mullenders and colleagues highlighted the importance of bladder tumor-PDO biobanks in disease modeling, revealing that organoids retained many common bladder cancer mutations like *TP53* and *FGFR3* [48].

A patient-derived xenograft (PDX)-derived organoid biobank was created for prostate tumors. The organoids preserved the tumor heterogeneity, lineage markers and transcriptome. In addition, *BRCA2*^-/-^ PDX-derived organoids were sensitive to olaparib (PARP inhibitor), similar to what was observed in patients [49].

### 3.4. Nervous System and Head and Neck District

Few PDO biobanks have been created for tumors of the nervous system and head and neck district. Recently, two research groups established a PDO biobank from glioblastoma and glioma primary samples, respectively [50,51]. Both, glioblastoma and glioma PDOs maintained the inter- and intra-tumoral transcriptomic and genomic heterogeneity compared to the parental tumor [50,51]. Interestingly, glioblastoma PDOs were co-cultured with 2173BBz CAR-T cells designed to specifically react with cells expressing EGFRvIII (a common variant found in glioblastomas) revealing that EGFRvIII^+^ cells were specifically targeted and killed by 2173BBz CAR-T cells [50]. As for the head and neck district, a PDO biobank was created from primary and recurrent nasopharyngeal carcinoma, showing that all PDOs were *EBER* (EBV-encoded small RNA) positive and *CK7* negative, thus retaining features of the parental tumors, including EBV infection status and clinical characteristics. In addition, recurrent nasopharyngeal organoids showed higher expression of BMI-1, CD44 and CD133 stem cell markers [52].

### 3.5. Respiratory District

Kim and colleagues created a PDO biobank from patients with advanced lung adenocarcinoma, revealing that PDOs retained driver mutations of original tumors and were able to predict a mutation-specific clinical drug response of patients [53]. Furthermore, they showed the efficacy of two drugs against *ERBB2* exon 20 insertion and *RET* fusion molecular targets [53]. Another research group established a living biobank of PDOs from patients with non-small cell lung cancer (NSCLC), comparing the efficacy of different anti-cancer natural compounds between PDOs and cell lines, allowing for the use of NSCLC PDOs for high-throughput screening [54].

## 4. Culture Media for Long-Term Expansion of PDOs

**Figure 4:**
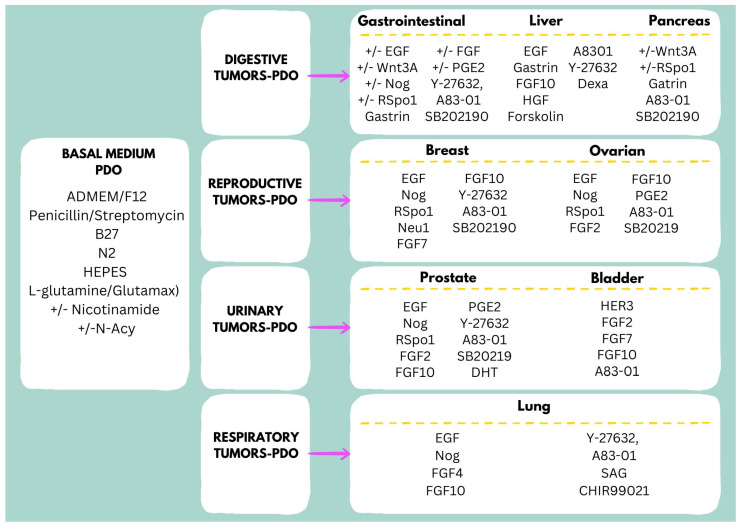
Tumor-specific culture media for long-term expansion of PDOs. Search words: organoid, organoids culture media, gastrointestinal/liver/pancreas/breast/ovarian/prostate/bladder/lung organoids (databases: PubMed, Google Scholar).

Choosing appropriate conditions for long-term culture of PDOs requires careful consideration. Extensive experimentation is required to optimize a specific culture protocol that allows PDOs to retain the morphological and genetic properties of the parental tumor (or paired healthy tissue) over multiple passages. Since PDO culture media substitute for factors provided by the tumor niche, their formulation, often combined with an extracellular matrix (ECM), must be tailored to the specific cancer type [67]. Indeed, beyond a shared basal medium, each tumor type requires a specific combination of cytokines and growth factors (Figure 4) [67,68]. Moreover, the mutational profile of a tumor may influence culture medium requirements, even among tumors of the same type [69]. Generally, the culture media for healthy tissue-specific PDOs establishment require less optimization, as they lack those mutations that could influence the different formulations of growth media.

Long-term culture of gastric tumor-PDOs needs a generic basal medium composed of ADMEM/F12 (advanced Dulbecco’s modified Eagle medium with nutrient mixture F12 Hams), antimicrobial reagent, B27, N2, HEPES and L-glutamine (or Glutamax), supplemented with EGF, noggin, R-spondin 1, gastrin, FGF-10, FGF-basic, Wnt3A, prostaglandin E2 (PGE2), Y-27632 (Rock inhibitor), nicotinamide (and/or N-acetylcystenine), A83-01 (TGF-β type I receptor inhibitor), SB202190 (p38 MAPK inhibitor) and HGF (exclusively for cholangiocarcinoma organoids) [27,70].

The culture medium for intestinal tumor-PDOs shares several common factors with that of gastric tumor-PDOs. Many of the key optimization experiments for intestinal PDOs were conducted by Hans Clevers’ group, who studied tumors with different mutational profiles as well as matched healthy mucosa. Their findings indicated that long-term expansion of PDOs from healthy intestinal or colonic mucosa requires a basal medium supplemented with Wnt3A (recombinant human Wnt-3A or Wnt-3A conditioned medium), EGF, noggin, R-spondin 1, gastrin, nicotinamide, A83-01 and SB202190 [2]. Of note, Wnt3A, R-spondin 1, noggin, and EGF are supplemented according to the specific mutational profile of tumors, while A83-01 and SB202190 are generally needed by all PDOs derived from intestinal/colorectal tumors [2,69]. Interestingly, the addition of interleukin-22 is necessary to guarantee the formation of Paneth cells in intestinal-PDOs [71].

Broutier and colleagues reported optimal conditions for the long-term culture of liver carcinoma PDOs, including hepatocellular carcinoma (HCC), cholangiocarcinoma (CC) and combined HCC/CC (CHC) tumors [10]. They used a basal medium supplemented with gastrin, EGF, FGF10, HGF, forskolin, A8301, Y27632 and dexamethasone for HCC-PDOs. Interestingly, noggin, R-spondin 1 and Wnt3A are not required for the isolation of CC PDOs, whereas R-spondin 1 maintains cell growth in CC PDOs [10].

PDAC-PDOs differ from others in that they do not always require EGF or Wnt enhancers in the culture medium [72]. Indeed, distinct PDAC-PDO subtypes were identified, including a Wnt-non-producing subtype that needed Wnt ligands, a Wnt-producing subtype that autonomously secreted Wnt ligands and an R-spondin-independent subtype that grew in the absence of Wnt and R-spondin 1 [72].

Several other tumor-PDOs do not always require Wnt ligands in the culture media to enhance growth efficacy. For breast carcinomas, it was shown that neuregulin 1 (ligand for ERBB3 and ERBB4 tyrosine kinase receptors), but not Wnt3A, is indispensable for the isolation and long-term culture and expansion of breast cancer PDOs [38]. Moreover, the addition of Y-27632 improved the PDO growth while SB202190 (>1 μM) decreased it. Notably, the presence of EGF (>5 ng × mL^−1^) enhanced proliferation, but caused PDOs to acquire a two-dimensional appearance by moving toward the bottom of the plate [38]. Ovarian carcinoma-PDOs can be efficiently cultured without Wnt3A in a basal medium supplemented with R-spondin 1, noggin, EGF, FGF-10, FGF2, nicotinamide, N-acetylcysteine, prostaglandin E2, SB202190 and A83–01 [73].

For long-term cultivation of prostate cancer-PDOs, the required medium is similar to that of ovarian carcinoma-PDOs, with dihydrotestosterone (DHT) and Y-27632 representing important additional factors [74]. The culture medium for bladder tumor PDOs is quite peculiar. Whyard and co-workers reported that supplementation of basal medium with a combination of HER3, FGF2, FGF7, FGF10, A83-01, N-acetylcysteine, and nicotinamide is essential for the long-term maintenance of bladder organoids [75]. Lung carcinoma PDO cultures require a basal medium supplemented with SAG (agonist of smoothened, part of the hedgehog signaling pathway), CHIR99021 (glycogen synthase kinase (GSK) 3 inhibitor), EGF, noggin, FGF10, FGF4, N-acetylcysteine, A8301 and Y27632 [76].

## 5. Challenges and Limitations in the Establishment of PDO Biobanks

Despite their growing use in translational research, PDO cultures have several limitations. The generation and maintenance of PDOs can be time-consuming and labor-intensive, with success rates varying depending on the tumor type and the quality of the primary tissue. Genetic drift or selective pressure during prolonged culture may also lead to discrepancies between the original tumor and the organoid, potentially affecting the validity of downstream analyses. Additionally, current PDO models often lack standardized protocols—for example, for cell culture media and extracellular matrix (ECM)—which limits reproducibility across laboratories. Furthermore, one major constraint is their inability to fully replicate the complex tumor microenvironment, including interactions with immune cells, blood vessels, and stromal components, all of which are critical to tumor progression and treatment response. Further details are reported in the following sections.

### 5.1. Sample Acquisition and Quality Control of PDO Culture Processes

PDOs are valuable tools for disease modeling, drug screening and development, biobanking, and other applications. Scientists have made significant progress in creating organoid biobanks by developing a range of effective organoid culture systems as described previously (Figure 3 and Figure 4).

The success rate for the establishment of PDO cultures depends on proper initial tissue handling, the processing protocols to isolate PDOs, the choice of culture media, and cryopreservation and storage.

However, despite substantial advances, the success rate for the establishment of PDO cultures and recovery after cryopreservation is still limited, posing a major challenge to the effective creation of living biobanks. As a result, quality control during PDO culture procedures has been a primary focus. The first step involves acquiring fresh tissue while maintaining its quality and viability. Obtaining high-quality, viable tissue samples is essential for accurate research, but variability in sample quality can occur due to differences in sample collection methods, processing and storage times [77,78].

Based on our experience, freshly resected tissues should be promptly placed in a preservation solution and transported to the biobank laboratory on ice. To avoid ischemic processes, the time between resection and management at the laboratory should be as short as possible. Organoid cultures can be generated directly from fresh tissues or, alternatively, from frozen primary tissues, as first described by Walsh and colleagues in 2016 [79]. PDOs are stored in cryopreservation solution at −80 °C overnight and then immediately transferred into liquid nitrogen for long-term storage. This process influences the success rate of resuscitation. Several studies have shown that tissues directly frozen after resection can be effectively used for various applications, such as establishing 2D and 3D cultures, ex vivo cultures, and single-cell RNA sequencing, yielding results comparable to those obtained from freshly analyzed material [80,81,82]. Recently, Jiang et al. showed that gastric cancer PDOs generated using collagenase and without the ROCK inhibitor Y-27632 were more successful than those constructed using Liberase TH, TrypLE Express, or EDTA digestion. Notably, EDTA digestion had a 50% lower success rate than other methods (*p* = 0.04), highlighting that variation in success rates is influenced by methodological differences [83].

### 5.2. Standardization of PDO Culture Processes

#### 5.2.1. Extracellular Matrix (ECM)

When transported into the biobank laboratory, following removal of non-epithelial parts and impurities, such as red blood cells, the tissue is minced and digested both mechanically and enzymatically. Finally, isolated cells are resuspended in an extracellular matrix (ECM), typically Matrigel, a protein matrix from the reconstituted basement membrane harvested from the Engelbreth–Holm–Swarm mouse sarcoma, which is widely used to mimic the ECM [84,85].

The use of Matrigel, however, has several limitations that produce inconsistent results in PDO cultures. Batch-to-batch variability, potential risk of immunogenic reactions, scalability and reproducibility are the most reported limitations of this natural, animal-derived ECM, thus making the production of large quantities of Matrigel with consistent quality and composition necessary for the maintenance of experimental reproducibility extremely challenging.

To overcome these limitations, synthetic alternatives to natural ECM such as hydrogel, synthetic polymers and peptide-based ECMs were developed [86,87,88,89,90]. Compared to Matrigel, synthetic ECMs can be precisely engineered with defined compositions, leading to high reproducibility and consistency across experiments. In addition, they have reduced immunogenicity, they are customizable and can be produced in large quantities with consistent quality, making them suitable for high-throughput applications and clinical scalability. Last, cost and availability should be considered. Synthetic ECMs can often be produced more cost-effectively and in larger quantities than natural ECMs, which may be important for large-scale studies and commercial applications. Indeed, Carpentier and colleagues found that synthetic gelatin-based hybrid hydrogels, including polyisocyanopeptides enriched with laminin–entactin complex (PIC-LEC) and gelatin methacryloyl (GelMA) provided a more defined and reproducible matrix compared to Matrigel for liver organoid culture [91]. Furthermore, in a co-culture model of gastrointestinal PDOs and dendritic cells, a synthetic hydrogel improved chemotactic motility of monocyte-derived dendritic cells compared to Matrigel. This enhancement facilitated better immune cell–epithelial interactions, demonstrating the potential of synthetic ECMs in complex tissue models [92].

Recently, decellularized porcine small intestinal ECM has been described as a natural alternative to Matrigel [89,93]. This approach involves removing cellular components from intestinal tissue, leaving behind the native ECM scaffold. Giobbe et al. demonstrated through transcriptomic and proteomic analysis that decellularized ECM maintained a proteomic signature of endoderm tissue, enriched with key ECM proteins essential for organoid formation, and thus retained the biochemical and structural properties of the native tissue. The decellularized ECM supported the formation and growth of human organoids from the small intestine, liver, stomach, and pancreas, and supported in vivo organoid growth [93]. These studies highlight the significant impact of ECM choice on organoid culture outcomes. While Matrigel has been widely used and continues to be used, likely due to standardized application protocols published in the literature, its undefined composition and variability pose limitations. Synthetic ECMs offer defined, reproducible, and tunable alternatives that can enhance organoid development, functionality, and scalability, making them valuable tools for research and therapeutic applications.

#### 5.2.2. Cell Culture Media

Long-term culture of PDOs requires a medium, the composition of which usually depends on the type of parental tissue and genetic profile (Figure 4) [13,94]. However, the genetic profile may not be assessed at the initial establishment of a PDO culture, thus requiring an optimization step where different combinations of growth factors are screened to guarantee a high success rate. Most importantly, literature research reveals that PDO culture methods vary significantly between research teams, raising concerns about the consistency of results and pointing to the necessity of setting up standard experimental procedures to be shared among scientists worldwide, since differences in medium composition can significantly affect experimental results [95]. Indeed, pharmacokinetic studies demonstrated that the gene and protein expression levels of most pharmacokinetic-related enzymes and transporters in human intestinal organoids is influenced by the composition of the culture medium [96].

Similarly, Wilson and colleagues showed that a Wnt3a-containing conditioned medium is optimal for the growth and survival of human colon organoids, in contrast to medium supplemented with recombinant Wnt3a alone, which does not support long-term survival due to decreased *LGR5* and *Ki67* expression. Furthermore, they found that removal of Wnt-3a, noggin and R-spondin growth factors resulted in the highest level of cell differentiation and in distinct epithelial phenotypes [97]. Furthermore, the presence of RSPO-1 in cell culture media influenced the Wnt–*pS6R* negative feedback loop in both non-*APC* (CRC-normal mucosae) and *APC*-mutated (familial adenomatous polyposis—normal mucosae) PDOs, suggesting that the composition of PDO culture media should be carefully considered when studying the interplay of molecular pathways [98].

Accordingly, Hogenson et al. described a profound influence of the culture medium on the phenotype of gastrointestinal cancer PDOs, with a significant difference in response to standard-of-care chemotherapies, distinct morphologies, and transcriptomes between media within the same PDO cultures [99].

#### 5.2.3. Tissue Microenvironment and Vascularization

Most PDOs consist solely of the epithelial layer and lack the physiological microenvironment, including components like the muscle layer, stromal cells, immune cells, vascular endothelial cells, and the nervous system. As a result, they have limited utility in emulating complex pathophysiological phenotypes, thus restricting research on immunotherapy and anti-vascular treatments. In addition, in the conventional method of organoid formation (dome-based and submerged) first described by the Clevers group [1] and widely adopted worldwide, nutrient supply and waste removal rely on passive diffusion. As PDOs grow and become more metabolically demanding, the limited diffusion of nutrients and oxygen into the inner regions of the 3D scaffold leads to the development of a necrotic core with significantly reduced cell viability, which eventually spreads throughout the entire ECM construct [100]. Recently, new technologies have been developed to overcome these limitations [101,102]. Additional insights on these innovative techniques, including co-cultures, organoids-on-a-chip, multi organoids-on-a-chip, air–liquid interface, and bioreactors are reported in the following section.

## 6. Future Perspectives and Solutions

**Figure 5:**
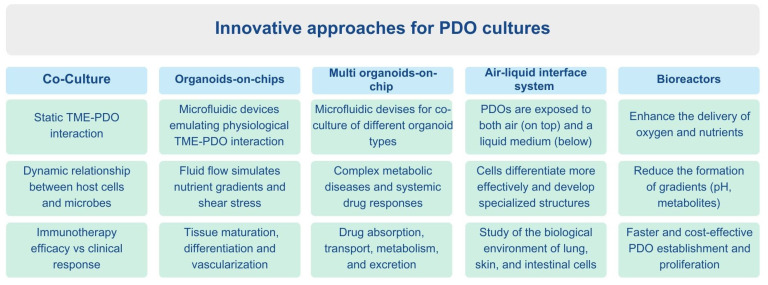
Overview of the main features related to innovative approaches for PDO cultures.

The absence of a tissue or tumor microenvironment (TME) in PDO cultures is a major limitation for drug-response studies that aim at improving personalized treatment therapies. This gap hampers the effectiveness of PDO biobanks. To overcome this, researchers have developed various methods to better mimic the in vivo physiological or pathological environment in PDO cultures, including co-cultures, organoids-on-a-chip, multi organoids-on-a-chip, air–liquid interface and bioreactors. An overview of the main features relative to these innovative approaches for PDO cultures is reported in Figure 5.

### 6.1. Co-Culture Systems

Co-cultures can partially replicate the TME by incorporating several cell types, such as immune cells, fibroblasts, or endothelial cells, as well as microorganisms [103,104,105]. Luo et al. developed a TME model using CRC PDOs embedded in a 3D hyaluronan–gelatin hydrogel and co-cultured with patient-derived cancer-associated fibroblasts (CAFs). In the absence of growth factors added to the co-culture, CAFs supported CRC PDO proliferation and restored biological pathways missing in PDO cultures but present in patient tissues [104]. Recently, several protocols for intestinal organoid co-cultures with microbes have been published [106,107,108]. Indeed, when co-cultured with microbes, these PDOs can be exposed to the same factors that would be present in a living organism, such as microbial metabolites, signaling molecules, and direct microbial interactions. This configuration helps to explore the dynamic relationships between host cells and microbes, shedding light on the roles of specific bacterial strains in health and disease. Tumor PDO–immune co-culture models have been created to study tumor-specific immune interactions and their possible therapeutic infringement [109,110,111,112]. Interestingly, Votanopoulos et al. showed that the response of the immune-enhanced patient tumor (lymph node/melanoma) organoids to immunotherapy was similar to the corresponding clinical response in 85% (6/7) of patients studied [109]. Furthermore, a co-culture model of T cells and midbrain organoids was developed to investigate neurodegeneration in Parkinson’s disease [113].

### 6.2. Organoids-on-Chips

Recently, the integration of organ chip technology with organoids has resulted in the creation of “organoids-on-chips,” which are microfluidic devices emulating organotypic models that more closely resemble physiological conditions [114,115,116,117]. As an emerging technology, organoids-on-chips can guide stem cell differentiation and organoid establishment within a controlled cellular microenvironment. Indeed, these chips integrate biological and physical components to support organoid culture and allow for precise manipulation of the conditions that influence organoid growth. Organoids-on-chips are usually made of glass, thermoplastics or of a combination of silicon mold and polydimethylsiloxane (PDMS) material with channels that allow fluid flow essential for organoid growth [118,119]. These channels simulate tissue environments and apply shear stress, influencing cell differentiation and vascularization. The chip also includes an extracellular matrix (ECM) scaffold that supports 3D cell growth and organoid formation. Stem cells or organ-specific progenitor cells are seeded onto the chip, where they form organoids, and endothelial cells may be added to create vascular structures [120,121]. Microfluidic systems control fluid flow to simulate nutrient gradients and shear stress, promoting tissue maturation [122].

Vascularization is crucial for organoid maturation in vitro, as it mimics the vascular networks in vivo that support organ development by providing nutrients and oxygen. To address nutrient diffusion limitations and improve organoid maturity, integrating vascular networks with organoids-on-chips is essential. Fluidic shear stress (FSS) has been shown to enhance vascularization by regulating endothelial cell functions, as demonstrated in kidney organoids on a millifluidic chip [123,124,125]. Homan et al. demonstrated that high FSS activated endogenous angiogenic pathways, thereby increasing the abundance of the vasculature, tissue functionality, and maturity of kidney organoids [125]. Furthermore, functional readout systems could help control organoid culture and improve understanding of the effect of the microenvironment on organoid development. Integrating multiplexed biosensors, like oxygen sensors and electrical arrays, offers potential solutions [126,127]. For example, Zhang et al. developed a modular organoid model with integrated sensors for continuous monitoring of organoid behavior and biochemical parameters [128]. Similarly, Schuster et al. created an automated microfluidic platform for real-time analysis of tumor organoid responses to drug treatments, advancing high-throughput drug screening and personalized therapy [129].

### 6.3. Multi Organoids-on-Chip

To mimic the complex physiological processes of drug absorption, transport, metabolism, and excretion, multi-organoid systems can be created by co-culturing different organoid types in compartmentalized microenvironments using organ chip technology. For example, a multi-layered organ chip has been developed to co-culture liver and heart organoids, providing a platform for studying systemic diseases and advancing drug development [130]. A liver-islet organoid-on-a-chip system has also been established to study diabetes mellitus, recapitulating the features of type 2 diabetes, and allowing the testing of therapeutic effects of drugs such as metformin [131]. Other organoid-on-a-chip systems, such as retinal and kidney tubuloids-on-chips, have been developed to study eye diseases and kidney functions, respectively [132,133]. These platforms enable the study of complex metabolic diseases and systemic drug responses.

### 6.4. Air–Liquid Interface System

Another cell culture method that aims to maintain the biological environment of cultured cells is the air–liquid interface (ALI) system. This specialized system allows epithelial cells to proliferate and differentiate, closely mimicking in vivo conditions [134]. ALI cultures were mainly developed for airway cell culture systems, since cells are seeded onto a porous membrane or scaffold that allows them to be exposed to both air (on top) and a liquid medium (below) [135,136]. This approach is often used to study cells that are naturally exposed to these two environments, such as lung, skin, and intestinal cells. Under these conditions, cells differentiate more effectively and often develop specialized features such as cilia, mucus production, or the formation of tight junctions, depending on the tissue type [137,138].

Kim et al. developed an ALI culture model using human lung organoids, which demonstrated a more than five-fold increase in ciliated cell production compared to submerged culture methods. Through transcriptomic analysis, the study identified two key regulatory pathways—*HIF1A–VEGFA* and *CDKN1A*—as central drivers of epithelial cell differentiation in this model [139]. Similarly, several studies have described the generation of gastrointestinal, respiratory and renal PDOs based on ALI systems, highlighting that this approach preserves organoids’ structural integrity, epithelial cell organization and functionality, making them suitable to assess chemotherapy resistance and toxicological effects in a tumor microenvironment [140,141,142,143].

### 6.5. Bioreactors

Another system that provides a controlled environment for both 2D and 3D cultures is the bioreactor. Bioreactors offer advantages over static droplet-based cultures by enhancing the delivery of oxygen and nutrients, while reducing the formation of gradients for pH, metabolites, and dissolved oxygen [144,145,146]. To date, four main categories of bioreactors have been developed for organoid culture: stirred bioreactors (SBR), microfluidic bioreactors (MFB), rotating wall vessels (RWV), and electrically stimulating (ES) bioreactors [147]. Spinning bioreactors have proven effective for generating kidney and brain organoids [148,149,150,151], and a spinner flask-based approach has been developed for the large-scale production of intrahepatic cholangiocyte organoids [152]. Ovando-Roche et al. found that the use of bioreactors results in improved laminar stratification as well as an increase in the yield of photoreceptor cells bearing cilia in retinal organoids grown in bioreactors compared to those grown in standard culture conditions [153]. Recently, Ye et al. developed a miniaturized (50 mL Falcon vessel) spinning bioreactor (RPMotion) for the effective production of human epithelial organoids derived from the liver, intestine, and pancreas, reducing time, labor, and costs compared to traditional bioreactors with larger capacities (100–2000 mL). All types of organoids exhibited faster proliferation in the RPMotion bioreactor (5.2-fold, 3-fold, and 4-fold, respectively) compared to static cultures, while maintaining their organ-specific phenotypes [154].

## 7. Conclusions

Patient-derived tumor organoids (PDOs) offer powerful tools that more accurately recapitulate the histological, genetic, and functional characteristics of their parental tumors, allowing significant advances in precision oncology. Currently, several PDO biobanks covering a wide range of tumor types have been established, providing reliable physiological and pathological models for both basic research and translational clinical studies.

Looking ahead, addressing challenges such as the standardization of organoid culture techniques, improving reproducibility, and establishing robust quality control systems will be critical. The development of safe, accurate, and scalable PDO biobanks will not only facilitate high-throughput drug screening and personalized therapy but also enhance our understanding of tumor heterogeneity and treatment resistance mechanisms. These efforts should be prioritized and supported by the wider research and clinical community to fully realize the potential of PDOs in cancer research and patient care.

## Figures and Tables

**Table 1:** Overview of tumor and paired healthy tissue-specific patient-derived organoid biobanks: technical features, worldwide distribution and main translational and clinical applications. Search words: translational research, precision medicine, disease modeling, gastrointestinal/liver/pancreas/breast ovarian/prostate/bladder/lung/kidney tumor patient-derived organoid biobank (databases: PubMed, Google Scholar).

System or Body District	Organ	Number of Samples		Country	Diagnosis	Primary or Metastatic	Main Experimental PDOs Validation	Main Translational Applications	References
		Tumor	Paired Healthy						
Digestive	Colorectal	22	19	The Netherlands	Colorectal carcinoma	Primary	WGS; RNA-seq	High-throughput screening (in vitro)	[17]
Digestive	Colorectal	55	41	Japan	Colorectal carcinoma	Primary and metastatic	Histology, WGS, RNA microarray,	Disease modeling	[18]
Digestive	Colorectal	32	18	China	Early-onset colorectal carcinoma	Primary	MSI analysis, WES, WGS, RNA-seq, sc-RNA-seq, gene editing	Disease modeling	[19]
Digestive	Rectal	96	0	China	Rectal carcinoma	Primary	Histology, WES	Drug/radiation response prediction	[20]
Digestive	Colorectal	151	0	China	Colorectal carcinoma	Primary and metastatic	Histology, RNA-seq	Drug response prediction	[21]
Digestive	Colorectal	94	0	The Netherlands	Colorectal carcinoma	Primary and metastatic	RNA-seq	Disease modeling	[22]
Digestive	Colorectal	58	0	China	Colorectal carcinoma	Primary and metastatic	Histology, WES, RNA-seq, sc-RNA-seq	Drug response prediction	[23]
Digestive	Colorectal	34	21	China	Colorectal carcinoma	Primary	Histology, WES	Drug response prediction	[24]
Digestive	Colorectal	77	31	The Netherlands	Colorectal carcinoma	Primary and metastatic	WES	High-throughput screening (in vitro/in vivo)	[25]
Digestive	Colorectal	106	0	Germany	Colorectal carcinoma	Primary and metastatic	WGS, WES, RNA-seq	High-throughput screening, gene–drug response correlation	[26]
Digestive	Colorectal, gastroesophagus, bile ducts	110	0	The United Kingdom	Colorectal, gastroesophageal cancers and cholangiocarcinoma carcinoma	Metastatic	Histology, WGS, NGS, RNA-seq,	High-throughput screening (in vitro/in vivo)	[27]
Digestive	Stomach	46	17	China	Gastric tumor	Primary and metastatic	Histology, WES, RNA-seq	High-throughput screening, drug response prediction	[28]
Digestive	Liver	11	0	Switzerland	Hepatocellular carcinoma	Primary and metastatic	Histology, WES	Disease modeling, drug response prediction	[29]
Digestive	Pancreas	13	13	USA	Intraductal papillary mucinous neoplasms	-	Histology, WGS	Disease modeling	[30]
Digestive	Pancreas	31	0	Switzerland	Pancreatic carcinoma	Primary and metastatic	Histology, WGS, WES, RNA-seq	Disease modeling, high-throughput screening, gene–drug response correlation	[31]
Digestive	Pancreas	77	0	USA	Pancreatic ductal adenocarcinoma	Primary and metastatic	Histology	Drug response prediction	[32]
Digestive	Pancreas	10	7	USA	Intraductal papillary mucinous neoplasms	-	Histology, WGS, WES, RNA-seq	Disease modeling	[33]
Digestive	Pancreas	30	5	The Netherlands	Pancreatic ductal adenocarcinoma and distal cholangiocarcinomas	Primary and metastatic	Histology, WGS, RNA-seq	High-throughput screening	[34]
Digestive	Pancreas	10	0	The Netherlands	Pancreatic carcinoma	Not specified	Histology, WGS, RNA-seq	Disease modeling	[35]
Digestive	Pancreas, gallbladder, duodenum	25	0	Japan	Gastroenteropancreatic neuroendocrine neoplasms	Primary and metastatic	Histology, WGS, WES, RNA-seq,	Disease modeling	[36]
Reproductive	Mammary gland	13	14	The Netherlands	Breast carcinoma (TNBC, ER+/PR+, Her2+)	Primary and metastatic	Histology, imaging	Disease modeling	[37]
Reproductive	Mammary gland	168	0	The Netherlands	Breast carcinoma (TNBC, ER+/PR+ or ER+/PR-, Her2+)	Primary and metastatic	Histology, WGS, RNA-seq	Drug response prediction	[38]
Reproductive	Mammary gland	33	0	Italy	Invasive ductal and lobular breast carcinoma (TNBC, Her2+, Her2-)	Primary	Histology	Disease modeling	[39]
Reproductive	Mammary gland	11	0	China	Breast carcinoma (TNBC, Her2+, Luminal B)	Primary and metastatic	Histology	Drug response prediction	[40]
Reproductive	Mammary gland	38	0	The Republic of Korea	Breast carcinoma (TNBC)	Primary and metastatic	RNA-Seq	Disease modeling, high-throughput screening gene–drug response correlation	[41]
Reproductive	Mammary gland	87	0	USA	Invasive ductal and lobular breast carcinoma (TNBC)	Primary and metastatic	Histology, WGS, RNA-seq, sc-RNA-seq	Disease modeling	[42]
Reproductive	Cervix	12	6	The Netherlands	Cervical tumors	Primary	Histology, WES, RNA-seq	Disease modeling, high-throughput screening	[43]
Reproductive	Ovaries	76	0	The United Kingdom	High-grade serous ovarian carcinoma	Primary and metastatic	Histology, WES, RNA-seq	Disease modeling, drug response prediction	[44]
Urinary	Kidney	54	47	The Netherlands	Kidney tumors	Primary and metastatic	Histology, WGS, sc-RNA-seq	Disease modeling	[45]
Urinary	Bladder	22	0	USA	Urothelial carcinoma	Primary	Histology, WGS	Disease modeling, gene-drug response correlation	[46]
Urinary	Bladder	16	0	The Netherlands	Urothelial carcinoma	Primary	Histology, WGS	Disease modeling, gene-drug response correlation	[47]
Urinary	Bladder	53	0	The Netherlands	Urothelial carcinoma	Primary	Histology	Disease modeling, high-throughput screening	[48]
Urinary	Prostate	4	0	USA	Prostate cancer	Metastatic	Histology, WGS, WES, RNA-seq	Gene–drug response correlation, drug response prediction	[49]
Nervous	Brain	70	0	USA	Glioblastoma	Primary	Histology, WES, RNA-seq, sc-RNA-seq	Disease modeling	[50]
Nervous	Brain	33	0	USA	Glioma	Primary	Histology, gene sequencing	Disease modeling	[51]
Head and Neck	Nasopharynx	62	15	China	Nasopharyngeal carcinoma	Primary	Histology	Disease modeling	[52]
Respiratory	Lungs	84	0	The Republic of Korea	Lung adenocarcinoma	Primary	Histology, WES, RNA-seq	Gene–drug response correlation	[53]
Respiratory	Lungs	14	0	China	NSCLC	Primary	Histology, gene sequencing	High-throughput screening	[54]

Abbreviations: ER+/PR+: estrogen receptor-positive/progesterone receptor-positive breast carcinoma; ER+/PR-: estrogen receptor-positive/progesterone receptor-negative breast carcinoma; Her2+: human epidermal growth receptor 2-positive breast carcinoma; MSI: microsatellite instability; NSCLC: non-small cell lung cancer; PDO: patient-derived organoid; RNA-seq: RNA sequencing; sc-RNA-seq: single cell RNA-sequencing; TNBC: triple-negative breast carcinoma; WES: whole-exome sequencing; WGS: whole-genome sequencing.

## Data Availability

No new data were created or analyzed in this study. Data sharing is not applicable to this article.
